# Gd-EOB-DTPA-enhanced MR relaxometry for the detection and staging of liver fibrosis

**DOI:** 10.1038/srep41429

**Published:** 2017-01-27

**Authors:** Michael Haimerl, Kirsten Utpatel, Niklas Verloh, Florian Zeman, Claudia Fellner, Dominik Nickel, Andreas Teufel, Stefan Fichtner-Feigl, Matthias Evert, Christian Stroszczynski, Philipp Wiggermann

**Affiliations:** 1 Department of Radiology, University Hospital Regensburg, Regensburg, Germany; 2 Department of Pathology, University Regensburg, Regensburg, Germany; 3 Center for Clinical Trials, University Hospital Regensburg, Regensburg, Germany; 4 MR Applications Predevelopment, Siemens AG, Healthcare GmbH, Erlangen, Germany; 5 Department of Internal Medicine I, University Hospital Regensburg, Regensburg, Germany; 6 Department of Surgery, University Hospital Regensburg, Regensburg, Germany

## Abstract

Gd-EOB-DTPA, a liver-specific contrast agent with T1-shortening effects, is routinely used in clinical routine for detection and characterization of focal liver lesions and has recently received increasing attention as a tool for the quantitative analyses of liver function. We report the relationship between the extent of Gd-EOB-DTPA- induced T1 relaxation and the degree of liver fibrosis, which was assessed according to the METAVIR score. For the T1 relaxometry, a transverse 3D VIBE sequence with inline T1 calculation was acquired prior to and 20 minutes after Gd-EOB-DTPA administration. The reduction rates of the T1 relaxation time (rrT1) between the pre- and postcontrast images were calculated, and the optimal cutoff values for the fibrosis stages were determined with receiver operating characteristic (ROC) curve analyses. The rrT1 decreased with the severity of liver fibrosis and regression analysis revealed a significant correlation of the rrT1 with the stage of liver fibrosis (r = −0.906, p < 0.001). ROC analysis revealed sensitivities ≥78% and specificities ≥94% for the differentiation of different fibrosis stages. Gd-EOB-DTPA–enhanced T1 relaxometry is a reliable tool for both the detection of initial hepatic fibrosis and the staging of hepatic fibrosis.

Liver fibrosis results from chronic damage to the liver, which is caused by, e.g., viral infection, alcohol abuse (ASH) or nonalcoholic steatohepatitis (NASH), in conjunction with the accumulation of extracellular matrix proteins that distort the hepatic architecture. The subsequent development of fibrotic scars, nodules of regenerating hepatocytes and destructive liver architecture defines cirrhosis, which is the final stage of chronic diffuse liver disease and is associated with portal hypertension and the risk for hepatocellular carcinoma, which is substantial cause of morbidity and mortality^1^ The progression and resolution of fibrosis is a complex process involving parenchymal and non-parenchymal liver cells, as well as infiltrating immune cells^2^. The increasing success of antiviral treatments in the blocking or reversing of the fibrogenic progression of chronic liver disease has established important principles and targets for antifibrotic drugs. Therefore, both the early detection and continuous monitoring of hepatic fibrosis have important clinical implications^3^. Liver biopsy is still considered the gold standard for the diagnosis and staging of liver fibrosis^4^. However, liver biopsies are poorly accepted by patients, may be prone to sampling errors and inter-observer variability, and are associated with risks of various complications, including infection and hemorrhage^5^. Therefore, non-invasive methods are required to assess the presence and severity of liver fibrosis.

Gadolinium ethoxybenzyl diethylene-triaminepentaacetic acid (Gd-EOB-DTPA; Primovist®, Bayer Healthcare, Berlin, Germany) is a paramagnetic hepatobiliary magnetic resonance (MR) contrast agent for T1- weighted imaging^6^. This agent is a gadolinium chelator that is taken up into the hepatocytes through organic anion-transporting polypeptides (OATP1B1/B3) in an ATP-dependent manner and is eventually excreted by the biliary pathway^7^. This is a property that Gd-EOB-DTPA shares with indocyanine green (ICG), which is commonly used to quantitatively assess liver function. Because the hepatic elimination of Gd-EOB-DTPA is dependent on the integrity of the hepatocyte mass, the quantification of Gd-EOB-DTPA uptake should represent the same aspects of liver function that can be assessed with ICG clearance tests^8^,^9^.

In this context, previous studies have demonstrated relationships between signal intensity measurements following Gd-EOB-DTPA administration with various clinical and biochemical parameters that are indicative of liver function^10^,^11^,^12^,^13^.

MR relaxometry has recently received increasing attention as a tool for the quantitative analyses of liver function that provides absolute parameters and is independent of many technical parameters and the manufacturer of the MRI machine^14^,^15^,^16^,^17^. The development of an accurate noninvasive method for assessing hepatic fibrosis could result in improved diagnostic abilities in terms of staging chronic liver disease^18^.

The purpose of this study was to assess the diagnostic performance of Gd-EOB-DTPA-enhanced T1 relaxometry in the staging of hepatic fibrosis in patients who have undergone liver biopsy or liver resection using histopathologic examination results as the reference standard.

## Results

The patient demographic variables according to fibrosis stage are summarized in Table 1. No significant differences were found in the demographic variables between the patients in the different fibrosis stages (F0–F4).

### T1 relaxometry measurements before Gd-EOB-DTPA administration (T1 pre) for the different stages of fibrosis

Before Gd-EOB-DTPA administration, there was no significant difference in the T1 relaxation times (T1 pre) between the patients without fibrosis(F0; 731.5 ± 73.1 ms) and patients with mild liver fibrosis (F1; 739.2 ± 72.3, p = 0.886). Furthermore no significant difference in T1 pre were observed between other adjacent stages of liver fibrosis (F2 = 672.3 ± 106.6 ms; F3 = 708.1 ± 160.1 ms, p = 0.172–0.548) with a tendency to lower T1 relaxation times for patients with liver cirrhosis (F4 = 582.3 ± 172.1 ms, p = 0.053).

**Figure 1:**
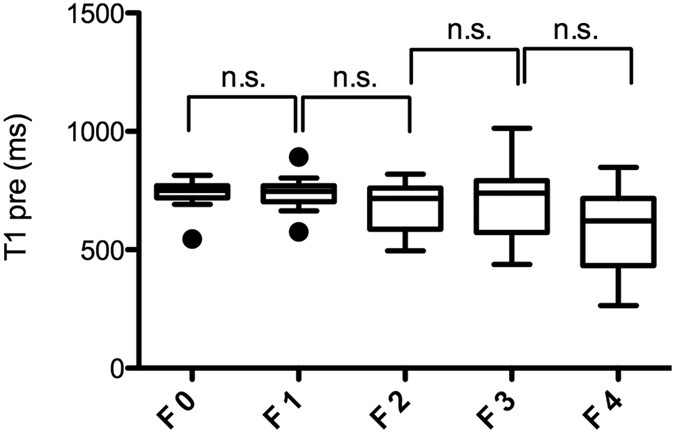
T1 relaxometry measurements in non-enhanced MRI according to the METAVIR score. Boxplots of the T1 relaxation times before GD-EOB-DTPA administration (T1 pre) in the patients with no liver fibrosis and the patients with liver fibrosis classified according to the METAVIR score (F0–F4). The data are provided as the mean T1 pre ± the standard deviation. n.s., non significant.

The corresponding boxplots are provided in Fig. 1.

### Gd-EOB-DTPA-enhanced T1 relaxometry measurements (T1 post, rrT1) for the different stages of fibrosis

**Figure 2:**
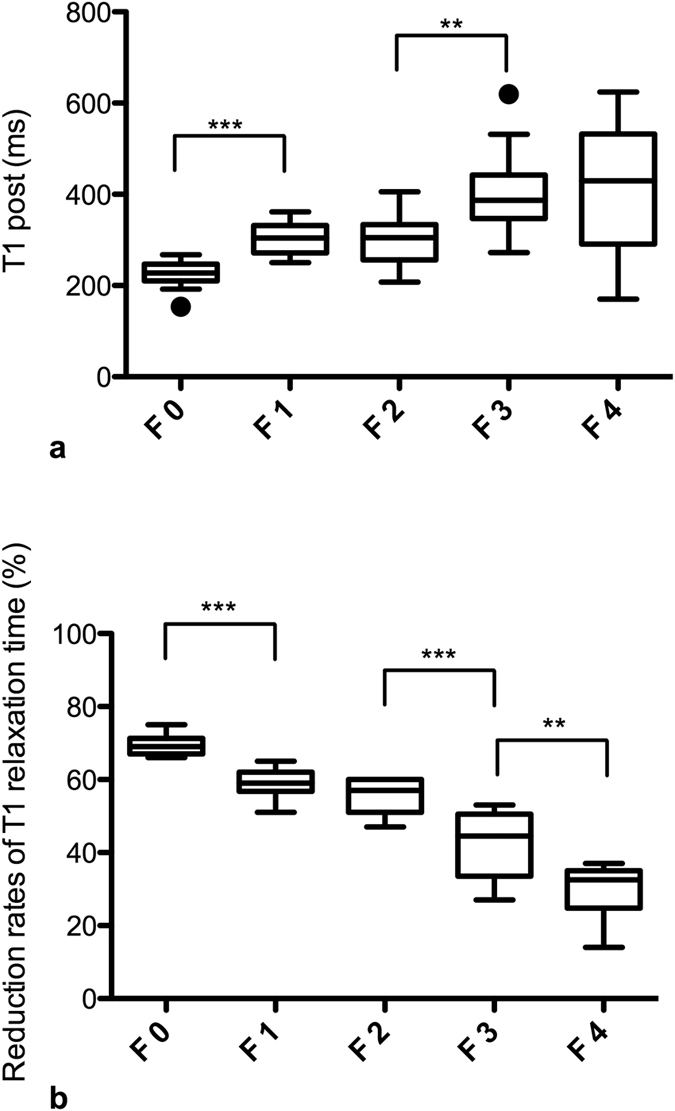
T1 post and reduction rates according to the METAVIR score. Boxplots of the T1 relaxation times 20 min after GD-EOB-DTPA administration (T1 post, Fig. 2a) and the respective reduction rates of the T1 relaxation time (Fig. 2b) in the patients with no liver fibrosis and the patients with liver fibrosis classified according to the METAVIR score (F0–F4). The data are provided as the mean T1 post and mean reduction rate ± the standard deviation. ***p < 0.001; **p < 0.01.

Significant differences in T1 post were observed between the patients without fibrosis (F0, T1 post, 224.3 ± 33.0 ms) and the patients with mild liver fibrosis (F1; T1 post, 302.1 ± 36.0 ms, p < 0.001) and between the patients with moderate (F2; T1 post, 300.6 ± 58.0 ms) and severe liver fibrosis (F3; T1 post, 403.6 ± 93.8 ms, p = 0.002). No significant differences in the T1 post values were observed between the other adjacent fibrosis stages (Fig. 2a, Table 2).

**Figure 3:**
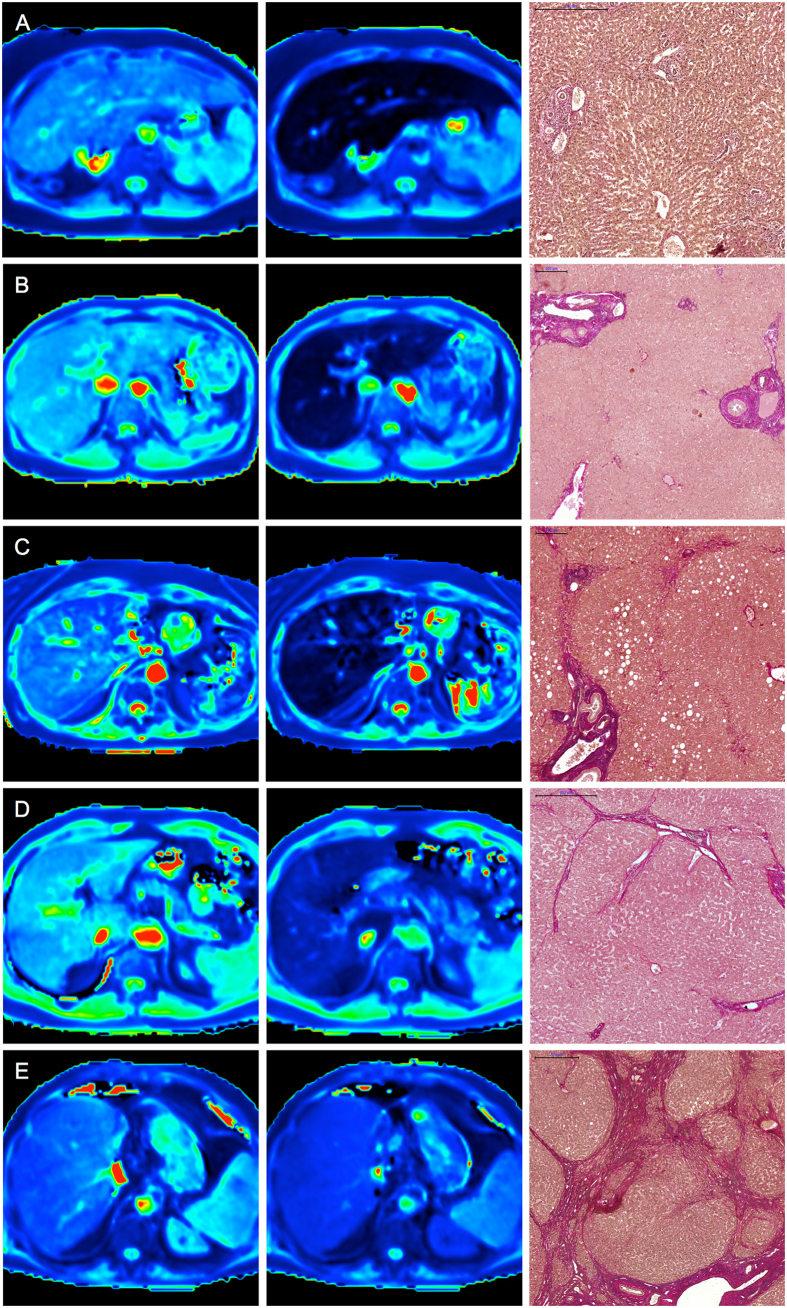
T1 maps and histopathological images. Color-coded T1 maps comparing the precontrast and hepatobiliary phases of a patient with normal liver parenchyma (F0, **A**) and those of patients with different stages of liver fibrosis (METAVIR stages F1–F4, **B–E**) with the corresponding EVG-stained histopathological images. The T1 maps calculated from the 3D VIBE sequences (TR 5.79 ms, TE 2.46 ms) with inline corrections for B1 heterogeneities were obtained before and 20 min after the administration of Gd- EOB-DTPA. The scale on the histopathology images represents 500 μm (**A,D,E**) and 200 μm (**B,C**), respectively. The reduction rates of the T1 relaxation times between the precontrast and Gd-EOB-DTPA-enhanced images were as follows: (**A**) no liver fibrosis (F0), rrT1: 0.71; (**B**) mild liver fibrosis (F1), rrT1: 0.63; (**C**) advanced liver fibrosis (F2), rrT1: 0.52; (**D**) severe liver fibrosis (F3), rrT1: 0.41; and (**E**) liver cirrhosis (F4), rrT1: 0.28.

Figure 2b provides boxplots of the rrT1 values for the patients with no liver fibrosis (F0) and the patients with liver fibrosis according to the METAVIR score classifications (F1–F4). In the comparison of the patients with no liver fibrosis (F0) and those with liver fibrosis (F1–F4), a decrease in the rrT1 in the cases of liver fibrosis was revealed as a constant decrease in rrT1 with the increasing progression of fibrosis (Figs 2b and 3, Table 2). Significant differences in the rrT1 values were observed between the patients without fibrosis (F0; rrT1, 69.4 ± 2.6) and those with mild liver fibrosis (F1: rrT1, 59.1 ± 3.7, p ≤ 0.001), between the patients with moderate liver fibrosis (F2; rrT1, 55.3 ± 4.6) and severe liver fibrosis (F3; rrT1, 42.2 ± 9.6; p < 0.001) and between the patients with severe liver fibrosis (F3) and liver cirrhosis (F4; rrT1, 28.8 ± 8.2, p = 0.004). However, there was no significant difference between the patients with mild and moderate liver fibrosis (F1, F2; p = 0.077). An analysis of the relationship between rrT1 and the histologic parameters in our study group revealed that rrT1 was strongly negatively correlated with fibrosis progression (r = −0.906, p < 0.001).

### Comparison of the different sample acquisition methods

Comparison of the patients who underwent liver resection with those who underwent needle biopsy revealed no significant differences regarding the rrT1 values of the fibrosis stages (p = 0.32). The patients who underwent liver resection (mean fibrosis stage: 2.0 ± 1.2) tended to exhibit higher rrT1 (52 ± 13%) and lower T1 post values (315.1 ± 96.9 ms) compared with the patients who underwent needle biopsy (mean fibrosis stage 2.2 ± 1.7; 47 ± 18%; 363.0 ± 120.2 ms); however, there were no significant differences between these two groups. Both groups exhibited a strong correlation between the rrT1 values and the histologic parameters (needle biopsy; r = −0.893, p < 0.001; liver resection; r = −0.874, p < 0.001)

### Diagnostic performance of the reduction rate of the T1 relaxation time

A ROC analysis was performed to differentiate the different stages of liver fibrosis based on the rrT1.

**Figure 4:**
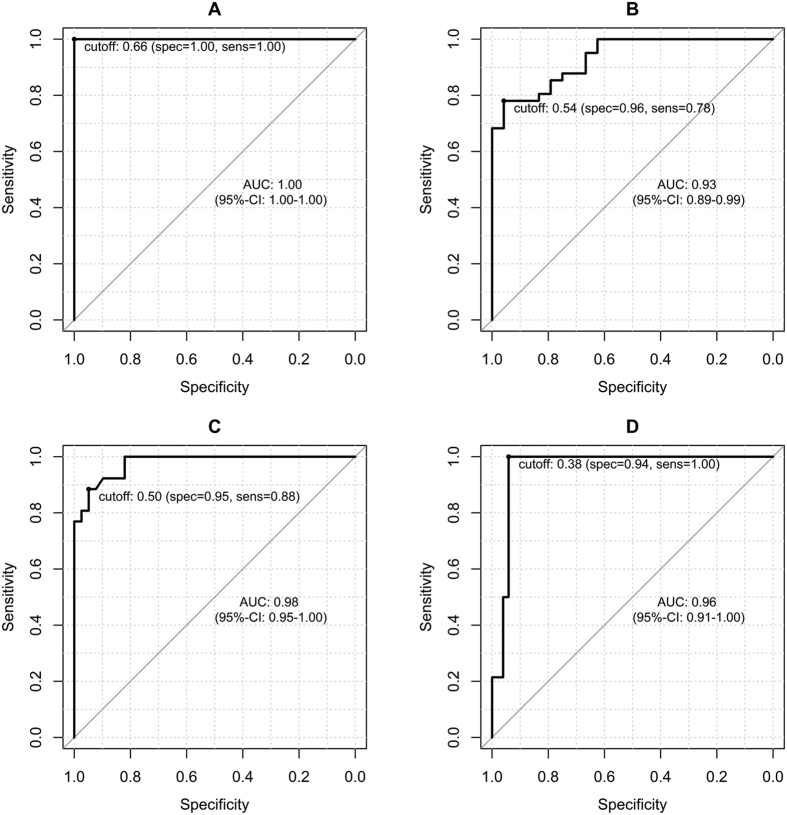
ROC analysis. Graphs showing the ROC curves for the reduction rates of the T1 relaxation times (rrT1) for the diagnoses of liver fibrosis in the patients with (**A**), METAVIR stage 1 or greater fibrosis (≥F1; rrT1, cutoff 0.66), (**B**), stage 2 or greater fibrosis (≥F2; rrT1, cutoff 0.54), (**C**), stage 3 or greater fibrosis (≥F3; cutoff 0.50), and (**D**), stage F4 fibrosis (rrT1, cutoff, 0.38).

Figure 4 provides the ROC curves according to the following four different fibrosis stage thresholds: F1 or greater (≥F1), F2 or greater (≥F2), F3 or greater (≥F3), and F4. The optimal cutoff values with the corresponding sensitivity and specificity values are provided in Table 3. The maximal diagnostic accuracy obtained using the rrT1 value was 1.0 for the diagnosis of fibrosis of stage 1 or greater (≥F1). The diagnostic accuracy for fibrosis stage 2 or greater (≥F2) was 0.93, and the diagnostic accuracy for fibrosis stage 3 or greater (≥F3) was 0.98. Finally, the diagnostic accuracy for liver cirrhosis (F4) was 0.96.

## Discussion

Hepatic fibrosis is currently considered to be a dynamic process that can regress to a recompensated phase in cases in which adequate treatment is provided at the appropriate time^19^. In the last few years, numerous antifibrotic agents have been developed, and important principles and targets for antifibrotic drugs have been established^3^. Thus, the early detection of hepatic fibrosis has important clinical implications, and a noninvasive, reproducible test is desirable. Currently, liver biopsy remains the gold standard for both the diagnosis and staging of liver fibrosis. However, liver biopsies are invasive procedures with consistently poor patient compliance. Furthermore, liver biopsies are prone to sampling errors and interobserver variability due to subjective morphological evaluations and are associated with risks of various complications, such as hemorrhaging and infection^5^,^20^.

Therefore, recent advances in magnetic resonance imaging have led to a growing interest in the optimization of MRI for the evaluation of diffuse liver disease. Such MR imaging methods include diffusion-weighted imaging, which depicts the restriction of water diffusion^21^,^22^; perfusion-weighted MRI^23^,^24^; and MR elastography. The latter technique offers direct insight into the parenchymal composition, stiffness and mechanical transformations of the liver and provides a high level of accuracy for the detection of all stages of liver fibrosis^25^,^26^,^27^. However, MR elastography remains expensive and not widely accessible for imaging routines for patients with liver diseases.

Together, these studies demonstrate that the mentioned MRI techniques are of limited use in the diagnosis and staging liver fibrosis because they generally focus on morphological or perfusion-related changes in the liver caused by liver fibrosis rather than the molecular changes of fibrosis itself 28.

Using non-contrast T1 maps, our results revealed no significant differences between the liver fibrosis METAVIR stages F0–F4 (p = 0.053–0.886).The characteristics of non-contrast T1 relaxation times in patients with and without liver fibrosis and cirrhosis have been controversially discussed in the current literature. Although some studies failed to identify any significant differences in the T1 relaxation times between patients with normal liver function and patients with liver fibrosis or cirrhosis on non-enhanced T1 maps^29^,^30^,^31^, prolonged T1 relaxation times have been reported and are most likely due to tissue remodeling in liver fibrogenesis, which is characterized by inflammation and edema^15^,^32^. However, the latter changes actually characterize the early stage of fibrous formation, which results in augmented hepatic water content and an increase in the ratio of free to bound water with a consequent prolongation of the T1 relaxation time^33^.

The advanced stages of liver cirrhosis induce both an increased deposition of paramagnetic macromolecules, i.e., collagen, manganese, and copper, and a lower total water content^2^,^34^,^35^. Both of these processes result in decreased T1 relaxation times and thus accord with the findings of the present study. However, non-enhanced MRI is unlikely to be an appropriate method for the detection and staging of liver fibrosis.

Gd-EOB-DTPA is a liver-specific, hepatobiliary contrast agent that produces both dynamic perfusion and liver-specific hepatobiliary MR images and thus combines the properties of an extracellular fluid contrast agent and a hepatobiliary agent^6^. The transport of Gd-EOB-DTPA in the hepatocytes is mediated by different transport systems that are located at the sinusoidal and canalicular membranes of the cell. The contrast agent enters the hepatocytes through two organic anion-transporting polypeptide transporters (OATP1B1 and OATP1B3), and it is excreted into the biliary system via the multidrug resistance protein 2 (MRP2)^36^. Both the expression and transport functions of these transporters are modified in diffuse liver diseases; thus, Gd-EOB-DTPA has been used to assess the severity of liver function impairment^7^,^37^. Impaired Gd-EOB-DTPA liver uptake may be caused by either a decrease in the number of normal, functioning hepatocytes or a decrease in gadoxetate disodium uptake by the hepatocytes due to the malfunctioning of the OATP1-MRP2 pathway^38^. Experimental studies with induced liver fibrosis in rats have demonstrated reduced OATP1 expression and upregulated MRP2 expression, both of which are associated with significant signal intensity loss following Gd-EOB-DTPA administration^39^,^40^.

Several studies have examined the relationships between signal intensity measurements following the injection of Gd-EOB-DTPA and several liver function scores, such as the Child-Pugh classification^13^, the ICG clearance test^17^,^41^, and the MELD score^12^. Common to all of these measures, hepatic parenchymal enhancement at the hepatobiliary phase or relative enhancement of the liver have been reported to be lower in patients with chronic liver diseases and consequently impaired liver function than in those with normal liver function. In this context, only a few studies have compared GD-EOB-DTPA-enhanced signal intensity measurements with histopathologic data to evaluate the utility of Gd-EOB-DTPA-enhanced MRI for the staging of liver fibrosis. Here, to quantify liver parenchymal enhancement of the liver-to-spleen ratio^42^, the corrected liver-enhancement ratios^43^,^44^,^45^, the relative enhancement^46^,^47^ and dynamic measurements^24^ were used. However, signal intensity measurements are defined on an arbitrary scale and do not reflect absolute, comparable values. These measurements can depend on a variety of technical parameters, e.g., the gain of the radiofrequency amplifier and the MRI manufacturer, and can considerably vary at each imaging time^32^. Conversely, the T1 relaxation time is an absolute, comparable value (unit: ms) that is unaffected by these different factors. Therefore, the T1 relaxation time as measured with 3D VIBE sequences is considered to be very useful for comparing pre- and postcontrast enhancements. To the best of our knowledge, this is the first study to compare Gd-EOB-DTPA-enhanced T1 relaxometry measurements at 3.0 T with histologically proven stages of liver fibrosis.

In this study, we demonstrated that gadoxetic acid–enhanced MR imaging with T1 relaxometry measurements represents a potential diagnostic tool for both the detection and staging of liver fibrosis.

In our study population, a constant decrease in rrT1 with the increased progression of fibrosis (F0–F4) was demonstrated. Notably, a highly significant difference (p < 0.001) was observed between the patients with no liver fibrosis and those with initial fibrosis of the liver parenchyma (F1). Using T1 relaxometry at 3.0 T as a diagnostic tool and histopathological evaluation as the reference standard, we correctly classified all patients (100%) without liver fibrosis (F0). Given that the fibrotic remodeling process is at least partially reversible, the distinctions between normal and non-fibrotic liver parenchyma and initial liver fibrosis (F1) play crucial roles in the screening of liver fibrosis, the monitoring of early fibrotic changes and the application of adequate therapy as early as possible^19^. Using signal intensity measurements, Watanabe *et al*. and Goshima *et al*. differentiated patients with no liver fibrosis and initial fibrotic changes; however, the patient group with no liver fibrosis (F0), as considered in the study by Watanabe *et al*., included only three patients with pathologically proven F0 and 15 patients who were assumed to have healthy livers^44^. Moreover, Goshima *et al*. did not include pathologically proven F0 stage patients and used a multivariate analysis that included the volumetric ratio of the liver to the spleen to define the healthy F0 stage^45^. Motosugi *et al*. and Feiers *et al*. were unable to differentiate between patients with no liver fibrosis and patients with initial fibrosis^43^. However, Feiers *et al*. only used liver biopsies for the classification of liver fibrosis, and this approach may be prone to sampling errors^47^.

We observed a consistent significant decrease in rrT1 with the progression of liver fibrosis with the exception of a decrease in rrT1 from mild (F1) to moderate (F2) liver fibrosis that was not statistically significant (p = 0.077). This reduced difference between the stages of early fibrosis (F1, F2) accords with previously published findings of comparisons of the stages of liver fibrosis based on both signal intensity measurements and MR elastography^44^,^48^. Additionally, histopathological evaluations have demonstrated that increases in fibrotic remodeling are less evident in early fibrosis compared to the differences observed between F2 and F3 and between F3 and F4^49^.

In the ROC analyses, the best diagnostic performance was obtained for the distinction between normal liver parenchyma without any fibrotic tissue (F0) and liver fibrosis (METAVIR stages ≥ F1) with an AUC of 1.0 at the cutoff value of 0.66 (sensitivity 100%; specificity 100%).

Our study has several limitations. First, the trial was a single-center study with a limited patient population. Second, our correlation between rrT1 and fibrosis did not consider elevated hepatic fat or iron. Increased hepatic fat might decrease T1pre due to short T1 relaxation time of fat to a higher extent than T1post in absolute but not in relative terms. Thus liver fat is canceled out in rrT1. Hepatic iron is assumed to have only a minor influence on rrT1 as the variable flip angle method is a FLASH-based technique with rather short TE. Third, our study included various types of fibrosis, such as fibrosis induced by alcohol, fibrosis due to long-term viral hepatitis, and a combination of both. Future studies should use homogenous patient populations because the etiology of the fibrosis influences the parenchymal changes and may thus influence the resulting T1 relaxation times. Further prospective analyses of the diagnostic accuracy should be performed in more homogeneous patient cohorts and with larger patient populations. Fourth, the performance of liver biopsies up to 3 months before or after MR imaging is another limitation of our study. However, because the mean rate of liver fibrosis progression, as expressed in stage-specific transition probability values, is estimated to be 0.085–0.120 per year, the changes during this 3-month period are expected to be negligible^50^. Furthermore, due to limited capability to undergo breathhold examinations in patients with advanced liver cirrhosis, approximately 10% of patients were unable to complete the full MRI protocol. Lastly, the absence of both previous reaction to MRI contrast media and renal failure has to be emphasized as a necessary condition to undergo Gd-EOB-DTPA-enhanced T1 Relaxometry.

Our study supports the conclusion that measuring T1 relaxation times via Gd-EOB-DTPA-enhanced MRI may be incorporated into the clinical routine as a liver imaging screening test for the detection of silent disease and the non-invasive definition of the stage of liver fibrosis.

## Material and Methods

### Patients

Approval from the local institutional review board of the University Hospital Regensburg was obtained, and this retrospective study was performed in accordance with the relevant guidelines and regulations. Written informed consent for the use of the tissue samples and MR images for scientific research was obtained from each patient included in the study. The retrospective analysis covered the period between August 2013 and August 2015 and included all patients with histopathologically examined liver samples from liver biopsies or liver resection and MR relaxometry (n = 134). Liver biopsies were collected as a part of active patient monitoring in cases of suspected or known liver disease, and liver resections were performed in cases of liver cancer and metastasis.

The underlying cause for all patients with chronic liver disease were as follows: infection n = 16 (hepatitis B, n = 9; hepatitis C, n = 7), alcoholic liver disease n = 12, mixed and other n = 13. 15 patients had secondary liver malignancies (colorectal cancer n = 11, breast cancer n = 1, prostate cancer n = 3). 8 patients had a cholangiocellular carcinoma, 1 patient had an adenoma.

For inclusion in the study, the patients were required to have undergone a histologic assessment of their liver fibrosis stage according to the METAVIR score and to have undergone a Gd-EOB-DTPA-enhanced MR imaging examination at 3.0-T with prototype T1 relaxometry sequences using variable flip angles that was performed within 3 months of the histologic assessment of liver. All patients were free of histories of previous reactions to liver-specific MRI contrast media and contraindications for either MRI (e.g., claustrophobia and pacemakers) or Gd-EOB-DTPA administration related to renal failure (defined as a glomerular filtration rate below 30 ml/min).

Forty-one patients were excluded from this study due to insufficient liver samples (tissue lengths shorter than 15 mm and/or less than ten visible portal tracts). Seventeen patients had images with respiratory motion artifacts or severe imaging artifacts due to surgical clips that prevented analyses of the T1 relaxometry images, and an additional 11 patients were excluded due to the inability to complete the full MRI protocol due to poor breath-holding. The final study population therefore consisted of 65 patients (41 men and 24 women; mean age, 57.5 ± 14.3 years).

### MR Imaging

All images were acquired with a clinical whole-body 3.0-T system (Magnetom Skyra, Siemens Healthcare, Erlangen, Germany) and a combination of body and spine array coil elements (an 18-channel body matrix coil and a 32-channel spine matrix coil) was used for signal reception. A T1-weighted volume-interpolated breath-hold examination (VIBE) sequence with fat suppression was used to image the entire liver prior to and 20 minutes after the intravenous administration of Gd-EOB-DTPA (0.025 mmol per kilogram body weight). The hepatobiliary contrast agent Gd-EOB-DTPA was administered via a bolus injection at a flow rate of 1 ml/s and flushed with 20 ml NaCl. In addition to the routine imaging protocol, T1 relaxometry of the liver was performed before (non-contrast) and 20 min after the administration of Gd-EOB-DTPA (Primovist; Bayer Healthcare, Berlin, Germany) (hepatobiliary phase) using a prototype technique based on a 3D spoiled-gradient echo sequence using variable flip angles (1°, 7°, and 14°). A B1 map of the whole liver was acquired for each patient prior to the T1 relaxometry measurements to improve the homogeneity of the T1 maps in the critical anatomical areas, and color-coded T1 maps were calculated inline. For the T1 relaxometry, a 3D VIBE sequence (TR 5.79 ms, TE 2.46 ms) was applied with three different flip angles, and a voxel size of 3.6 mm x 2.5 mm x 4.7 mm interpolated to 1.3 mm × 1.3 mm × 3.0 mm was used. Using the controlled aliasing in parallel imaging results in higher acceleration (CAIPIRINHA) as a parallel imaging technique with an acceleration factor of 4, the entire liver was covered during a breath-hold (17 s).

### Image analysis

Three regions of interest (ROIs) in the liver (two in the right lobe and one in left lobe) were manually placed in the T1 maps before and after the administration of Gd-EOB-DTPA at the MR scanner workstation (Skyra, Siemens, Erlangen, Germany) to exclude visible vessels, focal solid liver lesions and imaging artifacts. The mean T1 values for the three ROIs were regarded as the representative T1 values of the liver. Each ROI was a circle (the sizes of the ROIs ranged from 1.2 cm^2^ and 4.9 cm^2^) and was chosen to be as large as possible. The ROIs of identical size and shape were placed with the same imaging sequences in the T1 maps before and after the Gd-EOB-DTPA administration and the hilum of the liver was chosen as a landmark.

The reduction rate in the T1 values (rrT1) between the pre-Gd-EOB-DTPA and post-Gd-EOB-DTPA enhancement images was calculated as follows:


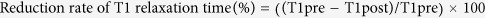


T1pre is the T1 relaxation time before the Gd-EOB-DTPA administration, and T1post is the T1 relaxation time 20 min after Gd-EOB-DTPA administration.

### Histopathological examination

For the histopathological examinations, liver biopsies (n = 25) and partial liver resections (n = 40) were used.

All samples were fixed in formalin and embedded in paraffin. Four-micrometer sections were cut vertically and mounted on glass slides. The sections were deparaffinized with xylene and ethanol and stained with hematoxylin-eosin (HE) and Elastica van Gieson (EVG) according to standard protocols. EVG staining was used to evaluate liver fibrosis. Collagen stained red, and the hepatocytes stained yellow.

An automatic needle device was used to obtain the liver samples from the non-tumorous liver biopsies. The length of each biopsy specimen was measured, and the numbers of portal spaces were assessed. The liver samples were included when the tissue length of 15 mm was exceeded, and more than ten portal tracts were visible. Two pathologists (K.U. and M.E.) who were blinded to the imaging results and the patient data independently reviewed the resection specimens and biopsies to evaluate the degrees of specific liver fibrosis. In cases of disagreements in terms of the common final judgment, additional microscopic analyses were performed together. Liver fibrosis was graded using the METAVIR scoring system, and patients were subdivided into the following 5 groups: patients with no liver fibrosis (F0; n = 10); mild liver fibrosis as defined by portal fibrosis without septa (F1; n = 14); moderate fibrosis as defined by portal fibrosis with few septa (F2; n = 15); severe fibrosis as defined by numerous septa without cirrhosis; (F3; n = 12) and liver cirrhosis (F4; n = 14).

### Statistical analysis

All statistical analyses were performed with IBM SPSS Statistics (version 23, Chicago, IL, USA) and R 3.2.1 software. The data are presented as the mean ± the standard deviation (SD). We used the non-parametric Mann-Whitney *U*-test for the independent variables and the Wilcoxon signed-rank test for the dependent variables in the comparisons between the groups. The relationships of the different histologic parameters with the rate of reduction in the T1 relaxation time were tested with nonparametric Spearman correlation coefficients. ROC analyses were performed to differentiate between the patient groups, and the optimal cut-offs were estimated according to the Youden indices. The estimates of the AUCs that corresponded with the 95% confidence intervals and the true classification rates are reported. All tests were two-sided, and values of p < 0.05 indicated significant differences.

## Additional Information

**How to cite this article**: Haimerl, M. *et al*. Gd-EOB-DTPA-enhanced MR relaxometry for the detection and staging of liver fibrosis. *Sci. Rep.* **7**, 41429; doi: 10.1038/srep41429 (2017).

**Publisher's note:** Springer Nature remains neutral with regard to jurisdictional claims in published maps and institutional affiliations.

## Figures and Tables

**Table 1:** Patient characteristics.

	All patients	F0	F1	F2	F3	F4
Age (years)	55.9 ± 14.8	48.2 ± 16.4	59.1 ± 14.3	58.5 ± 16.3	53.2 ± 14.8	57.5 ± 12.0
Sex						
Men, n (%)	41 (63)	2 (20)	7 (50)	10 (67)	9 (75)	13 (93)
Women, n (%)	24 (37)	8 (80)	7 (50)	5 (33)	3 (25)	1 (7)
Weight (kg)	81.1 ± 18.1	71.5 ± 17.4	81.7 ± 15.2	79.2 ± 20.4	83.8 ± 15.8	86.9 ± 19.8

The values indicate the mean ± the standard deviation. The stages of liver fibrosis are classified according to the METAVIR scores (F0, no liver fibrosis; F1, mild liver fibrosis; F2, moderate liver fibrosis; F3, severe liver fibrosis; F4, liver cirrhosis).

**Table 2:** T1 relaxation times of the liver in non-enhanced and Gd-EOB-DTPA-enhanced MRI and the reduction rates according to the liver fibrosis METAVIR stages F0–F4.

METAVIR	n	T1 pre (ms)	T1 post (ms)	rrT1 (%)
F 0	10	731.5 ± 73.1	224.3 ± 33.0	69.4 ± 2.6
F 1	14	739.2 ± 72.3	302.1 ± 36.0	59.1 ± 3.7
F 2	15	672.3 ± 106.6	300.6 ± 58.0	55.3 ± 4.6
F 3	12	708.1 ± 160.1	403.6 ± 93.8	42.2 ± 9.6
F 4	14	582.3 ± 172.1	418.3 ± 143.8	28.8 ± 8.2

The values indicate the mean ± the standard deviation.

T1pre: T1 relaxation time before Gd-EOB-DTPA administration.

T1post: T1 relaxation time 20 min after Gd-EOB-DTPA administration.

rrT1(%): reduction rate of the T1 relaxation time.

**Table 3:** ROC analysis. Cutoff values, sensitivities and specificities for the differentiations of the different fibrosis stages.

	Fibrosis stage ≥ F1	Fibrosis stage ≥ F2	Fibrosis stage ≥ F3	Fibrosis stage F4
rrT1 cutoff value	0.66	0.54	0.50	0.38
Sensitivity (%) (95% CI)	100 (0.90–1.0)	78 (0.62–0.89)	88 (0.70–0.98)	100 (0.68–1.0)
Specificity (%) (95% CI)	100 (0.59–1.0)	96 (0.79–1.0)	95 (0.83–0.99)	94 (0.81–0.98)
AUC (95% CI)	1.0 (1.0–1.0)	0.93 (0.89–0.99)	0.98 (0.95–1.0)	0.96 (0.91–1.0)
P value	<0.001	<0.001	<0.001	<0.001

rrT1, reduction rate of the T1 relaxation time; AUC, area under the ROC curve.
